# Nondisparate Survival of Non-Hispanic Black Women With Breast Cancer Despite Less Favorable Pathology: Effect of Access to and Provision of Care Within a Military Health Care System

**DOI:** 10.1089/heq.2022.0128

**Published:** 2023-03-10

**Authors:** Sarah Darmon, Leann A. Lovejoy, Craig D. Shriver, Kangmin Zhu, Rachel E. Ellsworth

**Affiliations:** ^1^ Murtha Cancer Center/Research Program, Uniformed Services University of the Health Sciences and Walter Reed National Military Medical Center, Bethesda, Maryland, USA.; ^2^ Chan Soon-Shiong Institute of Molecular Medicine at Windber, Windber, Pennsylvania, USA.; ^3^ Department of Surgery, Uniformed Services University of the Health Sciences, Bethesda, Maryland, USA.; ^4^ Department of Preventive Medicine and Biostatistics, Uniformed Services University of the Health Sciences, Bethesda, Maryland, USA.; ^5^ Henry M. Jackson Foundation for the Advancement of Military Medicine, Bethesda, Maryland, USA.

**Keywords:** breast cancer, disparities, pathology, survival, Black

## Abstract

**Introduction:**

Breast cancer mortality rates are 40% higher in non-Hispanic Blacks (NHBs) than in non-Hispanic White (NHWs) in the United States. All women treated within the Murtha Cancer Center at Walter Reed National Military Medical Center (MCC/WRNMMC) have health insurance and are provided multidisciplinary health care. Pathological factors and outcomes of NHBs and NHWs treated within the MCC/WRNMMC were evaluated to determine whether equal-access health care reduces disparate phenotypes and survival between the racial groups.

**Methods:**

Between 2001 and 2018, 368 NHB and 819 NHW women were diagnosed with breast cancer at MCC/WRNMMC. Differences between NHBs and NHWs in epidemiological and pathological characteristics were evaluated. Overall and breast cancer-specific 5- and 10-year survival rates were compared between races.

**Results:**

Compared with NHWs, NHBs were significantly more likely to have a body mass index ≥30 kg/m^2^, to be unmarried, to have tumors of higher grade, later stage, with lymph node metastases, and to be hormone receptor negative (HR^−^)/human epidermal growth factor receptor 2 positive (HER2^+^) or triple negative. After adjustment for demographic factors, NHBs remained significantly more likely to have tumors diagnosed at a higher grade and later stage, and to be HR^−^/HER2^+^ or triple negative. Neither 5- nor 10-year overall or breast cancer-specific survival differed significantly between the racial groups after adjusting for demographic and pathological variables.

**Discussion:**

Despite having tumors with less favorable pathological characteristics, overall and disease-free survival disparities were not observed for NHBs treated at MCC/WRNMMC. These data suggest that survival disparities of NHBs with breast cancer can be diminished with provision of quality care.

## Introduction

In the United States, breast cancer is a disparate disease in non-Hispanic Blacks (NHBs) compared with non-Hispanic Whites (NHWs). Historically, overall incidence rates were lower in NHBs than in NHWs, although in 2012, rates converged.^1^ NHBs are, however, significantly more likely to be diagnosed at a younger age, have higher stage and grade and larger size tumors, and be diagnosed with triple negative breast cancer (TNBC).^2^ In conjunction, mortality rates are 40% higher in NHBs than in NHWs,^3^ with the 5-year survival rates reported to be 81% for NHBs and 91% for NHWs.^4^

A number of factors have been proposed to account for disparate clinicopathology and survival for NHBs. For example, survival curves began to diverge between NHBs and NHWs in the mid-1980s, coincident with the use of endocrine therapies.^5^ Because NHBs are less likely to have estrogen receptor (ER)^+^ tumors, introduction of these early targeted treatments was beneficial to a smaller proportion of NHBs. Access to quality cancer screening and care can significantly contribute to less favorable outcomes in NHBs.^6^

A 2017 study found that implementation of public policy initiatives to decrease barriers to care instituted in Chicago, Illinois, resulted in a 20% reduction in survival disparity between NHWs and NHBs.^7^ Differences in health insurance may also affect survival disparities as lower stage-specific survival was detected in NHBs <50 years of age with ER^+^ tumors, or <65 years of age with ER^−^ tumors; no significant difference in survival, however, was detected for NHBs ≥65 years with either ER^+^ or ER^−^ tumors, suggesting that health insurance through Medicare reduces breast cancer disparities.^8^

The military health care system of the Department of Defense (MHS/DOD) is an equal-access health care system, in which all patients have health insurance and are provided with standardized cancer treatments. A study of women diagnosed with breast cancer between 1975 and 1994 found that although access to care was associated with improved overall survival for NHBs treated within the MHS/DOD compared with those in the United States general population, risk of death was 1.37 times higher in NHBs than in NHWs within the MHS/DOD.^9^

A second study that included women treated within the MHS/DOD 1980–1999 also found lower overall survival in NHBs than in NHWs.^10^ More recently, Rizzo et al evaluated outcomes in women with early stage breast cancer treated in the MHS/DOD and found no significant difference in overall survival.^11^ Importantly, none of these studies evaluated pathological characteristics or breast cancer-specific survival.

The Murtha Cancer Center at Walter Reed National Military Medical Center (MCC/WRNMMC), a member of the MHS/DOD, provides equal-access comprehensive breast care to all active-duty and retired service members and their beneficiaries. In this study, we investigated whether tumor pathology and overall and breast cancer-specific survival differed between NHBs and NHWs diagnosed between 2001 and 2018 at MCC/WRNMMC.

## Materials and Methods

The study participants were enrolled in the Clinical Breast Care Project (CBCP), MCC/WRNMMC. They were active-duty, veterans, or military beneficiaries of ages 18 years or older who were diagnosed with invasive breast cancer between 2001 and 2018. All enrollees voluntarily agreed to participate in the study and gave written informed consent. Demographic, pathological, and outcome data were collected with approval from the WRNMMC Human Use Committee and Institutional Review Board (WRNMMC IRB #20704). Only patients who self-described as NHBs or NHWs were included in this study.

### Data collection

Demographic and pathological data were available for 368 of 384 NHB and 819 of 850 NHW study-eligible women. Each patient was interviewed in person to collect data including family cancer and personal health histories, smoking and marital status, and education levels. A Charlson comorbidity index (CCI) was calculated for each patient using comorbidities existing before breast cancer diagnosis. Body mass index (BMI) was calculated based on the height and weight of the patient at diagnosis. Evaluation of surgical specimens for each patient was performed by a dedicated breast pathologist. Pathological data included anatomic tumor stage,^12^ size, grade,^13,14^ and lymph node status.

Biomarkers included in the analyses included ER, progesterone receptor, and human epidermal growth factor receptor 2 (HER2), with positivity assigned according to ASCO/CAP guidelines.^15,16^ Patient vital status was collected through December 31, 2020, from electronic health records.

### Statistical analyses

We first analyzed the distributions of demographic and pathological characteristics by race using a chi-square test. We then estimated the odds ratios of pathological factors adjusted for demographic variables, using either logistic regression or multinomial logistic regression. Overall and breast cancer-specific 5- and 10-year survival was compared between the racial groups using Kaplan–Meier plots and log-rank test statistics. Cox proportional hazards models were used while controlling for demographic and pathological factors. A *p*-value <0.05 was considered statistically significant. All statistical analyses were performed using SAS 9.4 (SAS Institute, Inc., Cary, NC).

## Results

The average age at diagnosis was 56.1 years for NHBs and 57.7 years for NHWs and no significant difference was detected by age group (Table 1). Education and smoking status were not significantly different between NHWs and NHBs. When compared with NHWs, NHBs were significantly more likely to have a BMI ≥30 kg/m^2^, be unmarried, and have a CCI <2.

**Table 1.** Distributions of Demographic Factors in NHB and NHW Women Diagnosed with Breast Cancer at MCC/WRNMMC, 2001–2018

		NHBs	NHWs	*p*
Age (years)	≥50	240 (65.2%)	558 (68.1%)	0.2168
	40–49	89 (24.2%)	199 (24.3%)	
	<40	39 (10.6%)	62 (7.6%)	
BMI (kg/m^2^)	<25	84 (24.7%)	280 (36.9%)	<0.0001
	25–29	112 (32.9%)	257 (33.9%)	
	≥30	144 (42.4%)	221 (29.2%)	
CCI	0	105 (29.7%)	221 (28.0%)	0.0304
	1	86 (24.3%)	151 (19.2%)	
	2	58 (16.4%)	184 (23.4%)	
	≥3	105 (29.7%)	232 (29.4%)	
Education	College degree	173 (55.1%)	401 (57.5%)	0.4692
	No college degree	141 (44.9%)	296 (42.5%)	
Marital status	Married	235 (64.0%)	658 (80.6%)	<0.0001
	Not married	132 (36.0%)	158 (19.4%)	
Smoking	Never	248 (68.1%)	553 (69.0%)	0.0694
	Former	79 (21.7%)	197 (24.6%)	
	Current	37 (10.2%)	52 (6.5%)	

BMI, body mass index; CCI, Charlson comorbidity index; MCC/WRNMMC, Murtha Cancer Center at Walter Reed National Military Medical Center; NHB, non-Hispanic Black; NHW, non-Hispanic White.

Breast tumors from NHBs were more likely to be diagnosed with lymph node metastases and to be of higher grade and stage than those from NHWs (Table 2). NHBs were more likely to have hormone receptor negative (HR^−^) tumors, including both HR^−^/HER2^+^and triple negative. After adjusting for demographic factors, NHBs remained statistically more likely to have tumors that were stage II, higher grade, and HR^−^/HER2^+^ or TNBC (Table 3).

**Table 2.** Racial Comparisons of Pathological Characteristics of Breast Cancer, MCC/WRNMMC, 2001–2018

		NHBs	NHWs	OR	Lower 95% CI	Upper 95% CI	*p*
Stage	Stage I	156 (42.4%)	451 (55.1%)	Reference			
	Stage II	153 (41.6%)	256 (31.3%)	1.728	1.318	2.265	<0.0001
	Stage III	46 (12.5%)	86 (10.5%)	1.546	1.035	2.311	0.0334
	Stage IV	13 (3.5%)	26 (3.2%)	1.445	0.725	2.882	0.2955
Grade	Low	77 (21.9%)	293 (36.7%)	Reference			
	Moderate	118 (33.5%)	294 (36.8%)	1.527	1.098	2.123	0.0118
	High	157 (44.6%)	211 (26.4%)	2.831	2.046	3.919	<0.0001
LN status	Negative	209 (59.2%)	518 (65.7%)	Reference			
	Positive	144 (40.8%)	270 (34.3%)	1.322	1.021	1.711	0.0342
HR/HER2	HR^+^/HER2^−^	216 (59.7%)	600 (74.3%)	Reference			
	HR^+^/HER2^+^	31 (8.6%)	63 (7.8%)	1.367	0.865	2.159	0.1804
	HR^−^/HER2^+^	40 (11.0%)	49 (6.1%)	2.268	1.452	3.541	0.0003
	TNBC	75 (20.7%)	95 (11.8%)	2.193	1.561	3.082	<0.0001

HER, human epidermal growth factor receptor 2; HR, hormone receptor; LN, lymph nodes; OR, odds ratio; TNBC, triple negative breast cancer.

**Table 3.** Odds Ratios for Pathological Factors in NHBs Compared with NHWs Diagnosed with Breast Cancer 2001–2018 After Adjustment

Response variable		Adjusted for demographics				Adjusted for demographics and cancer pathology			
		aOR	Lower 95% CI	Upper 95% CI	*p*	aOR	Lower 95% CI	Upper 95% CI	*p*
Grade	Low	Reference				Reference			
	Moderate	1.454	0.984	2.148	0.0602	1.522	1.003	2.308	0.0482
	High	2.542	1.725	3.746	<0.0001	2.333	1.448	3.759	0.0005
Stage	Stage I	Reference	.	.		Reference			
	Stage II	1.509	1.091	2.087	0.0129	1.070	0.683	1.678	0.7671
	Stage III	1.296	0.777	2.161	0.3209	0.884	0.437	1.785	0.7301
	Stage IV	1.073	0.424	2.720	0.8814	0.639	0.169	2.421	0.5103
LN status	Negative	Reference				Reference			
	Positive	1.228	0.894	1.686	0.2050	1.173	0.734	1.875	0.5044
HR/HER2	HR^+^/HER2^−^	Reference				Reference			
	HR^+^/HER2^+^	1.172	0.674	2.038	0.5745	0.892	0.478	1.664	0.7186
	HR^−^/HER2^+^	1.915	1.120	3.273	0.0175	1.339	0.710	2.523	0.3669
	TNBC	2.110	1.387	3.209	0.0005	1.315	0.795	2.176	0.2858

Controlled for age, BMI, CCI, education, marital status and smoking status, grade, lymph node status, stage, and HR/HER2 status.

aORs, adjusted odds ratios.

**FIG. 1.**
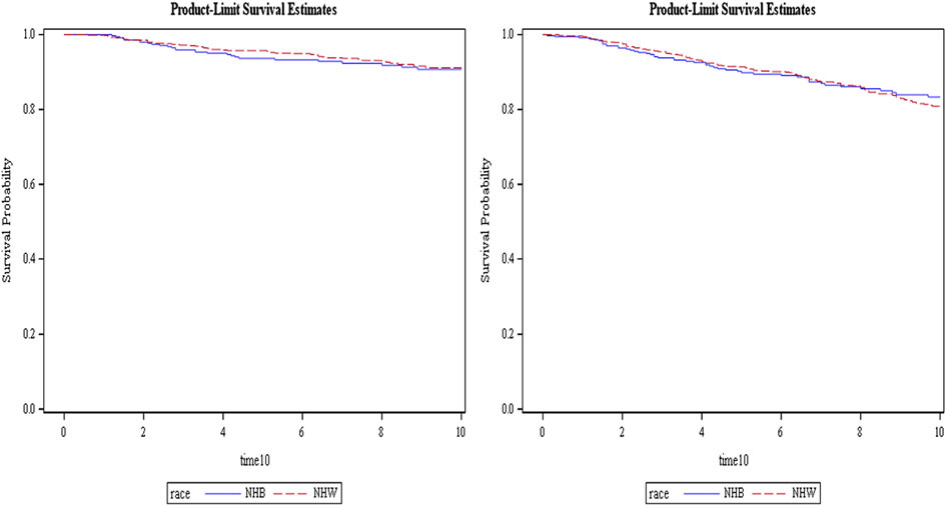
Ten-year breast cancer-specific survival (*left*) and overall survival (*right*). Log-rank *p*-values were 0.6193 and 0.6958 for breast cancer-specific and overall survival, respectively. Five-year survival curves are not shown, however, the *p*-values were 0.1725 and 0.5062 for breast cancer-specific and overall survival, respectively.

The average length of follow-up in this cohort was 9.0 years. Five- and 10-year overall and breast cancer-specific survival did not differ significantly between populations (Fig. 1). This remains true even after controlling for demographic and pathological factors. Breast cancer-specific survival rates were >90% for both NHBs and NHWs at 5 and 10 years (Table 4).

**Table 4.** Adjusted Hazard Ratios for NHBs Compared with NHWs with Breast Cancer in 5- and 10-Year Mortality, MCC/WRNMMC, 2001–2018

	NHBs	NHWs	aHR (95% CI)
5-Year			
Overall survival	91.0%	92.2%	1.1 (0.6–2.0)
Breast cancer-specific survival	94.1%	95.9%	1.1 (0.5–2.4)
10-Year			
Overall survival	87.0%	85.2%	0.8 (0.5–1.2)
Breast cancer-specific survival	92.2%	92.6%	0.8 (0.4–1.5)

Controlled for age, BMI, CCI, marital and smoking status, and education levels, stage, tumor characteristics (grade, type, size), node status, and HR (HER2, ER, and PR).

ER, estrogen receptor; PR, progesterone receptor.

## Discussion

Disparate survival in NHBs compared with NHWs with breast cancer has been recognized in a number of populations throughout the United States. A meta-analysis, using data reported from 1980 to 2005, found significantly higher risk of breast cancer-specific mortality for NHBs than for NHWs (mortality hazard: 1.19; 95% confidence interval: 1.10–1.29).^17^ For women treated at a single institution in Ohio between 2005 and 2014, NHBs had significantly lower overall and progression-free survival than NHWs,^18^ whereas NHBs diagnosed with breast cancer in Florida during 2010–2015 had 5- and 10-year mortality rates two times higher than those of their NHW counterparts.^19^

Using data from Surveillance, Epidemiology, and End Results (SEER) program, 40% higher mortality rates were observed for NHBs than for NHWs in 2015.^1^ Although these studies demonstrate disparate survival for NHBs, the SEER population represents a group with heterogeneous access to and provision of health care, which may contribute to the less favorable outcomes of NHBs.^20^

Having health insurance is one critical component in reducing cancer survival disparities.^21^ For example, individuals ≥65 years of age with Medicare coverage had significantly higher 5-year survival than those of age 60–64 years without insurance^5^ and, in a cohort of 563,497 women with breast cancer, matching for insurance reduced survival disparities between NHBs and NHWs by 37%.^22^ Coverage by a health insurance plan, however, is not sufficient to eliminate disparate outcomes, as Short et al found higher mortality rates for NHBs than for NHWs in a cohort of women with commercial health insurance.^23^

Similarly, Semprini and Olopade found that expansion of Medicaid under the Affordable Care Act did not improve mortality of NHBs with breast cancer.^24^ Insurance plans, however, differ by accessibility to and quality of care as shown by studies from Kaiser Permanente Southern California (KPSC), which provides integrative health care to its members. Lower mortality rates were observed for patients treated within the KPSC system compared with those with other forms of private health insurance,^25^ and breast cancer outcomes were not associated with race/ethnicity.^26^

The MCC/WRNMMC is similar to the Kaiser Permanente health care system in which all patients have insurance and are provided with integrative health care. All patients seen at MCC/WRNMMC are provided with comprehensive health care, regardless of rank or status. Standard coverage includes gynecological examinations with clinical breast examination and annual mammograms starting at age 40 years.

In addition, all women diagnosed with breast cancer meet with a multidisciplinary health care team, and services provided, such as surgery, including breast reconstruction, chemo- and radiation therapies, and psychological support, are covered, regardless of ability to pay. In conjunction, no significant differences were detected in overall or breast cancer-specific survival.

It is important to note that although survival disparities were not detected within this cohort treated at a military treatment facility, tumor pathology differences between NHBs and NHWs seen in the United States general population, including higher stage at diagnosis and higher prevalence of TNBC, were detected in our study population. Thus, the improved survival of NHBs treated at MCC/WRNMMC is occurring despite less favorable prognostics. Previous studies have shown that within the MHS/DOD, NHBs with regional stage tumors were less likely to receive chemotherapy and hormone therapy than NHWs,^27^ whereas time to surgery was longer and overall survival was worse for NHBs.^28^

Within the MCC/WRNMMC, however, time to surgery, breast cancer-specific survival,^29^ and uptake of germline genetic testing and election of risk-reducing surgeries did not differ between NHBs and NHWs.^30^ Additional studies evaluating provision of care and patient compliance within MCC/WRNMMC are needed to identify factors associated with decreased survival disparities.

This study does have several limitations. Our study was a hospital-based study of women treated exclusively at MCC/WRNMMC. As the only cancer center of excellence within the DOD, provision of care at MCC/WRNMMC may differ from that at other hospitals within the DOD. Thus, whether the lack of disparate outcomes detected in our study is generalizable to all the patients from MHS/DOD is unknown. Second, the majority of tumors in both populations were HR^+^, which have longer times to recurrence (5–20 years) and mortality (≥10-years) than HR^−^ tumors.^31,32^

Of note, the frequency of biomarker-derived subtypes in this study was similar to those from the SEER database,^4^ and, although the 5-year survival in NHWs was 96% in women from this study and 91% in those from SEER, the 5-year survival rate in NHBs was 94% in our study compared with 81% in those from SEER. Thus, a survival advantage for women treated at MCC/WRNMMC was detected at 5 years.

Continued monitoring of the MCC/WRNMMC cohort is critical to determine whether disparate survival rates diverge significantly after 10 years, especially for women with HR^+^/HER2^−^ breast tumors. Finally, although quality of life (QOL) after breast cancer diagnosis is crucial to the overall health of the breast cancer survivor, QOL data were not routinely collected from patients treated at MCC/WRNMMC. Thus, future studies are needed to determine whether QOL is disparate between NHB and NHW survivors.

## Conclusions

Despite having tumor characteristics associated with less favorable outcomes, survival did not differ between NHBs and NHWs treated within the MCC/WRNMMC. These data demonstrate that breast cancer survival disparities were mitigated within MCC/WRNMMC. Future studies that identify those elements of care that lead to comparable overall and breast cancer-specific rates of survival are critical to reducing disparate outcomes within not only the MHS/DOD but the U.S. general population as well.

## Abbreviations Used

aORs: adjusted odds ratios
BMI: body mass index
CCI: Charlson comorbidity index
ER: estrogen receptor
HER2: human epidermal growth factor receptor 2
HR: hormone receptor
KPSC: Kaiser Permanente Southern California
LN: lymph nodes
QOL: quality of life
MCC/WRNMMC: Murtha Cancer Center at Walter Reed National Military Medical Center
NHB: non-Hispanic Black
NHW: non-Hispanic White
ORs: odds ratios
PR: progesterone receptor
SEER: Surveillance, Epidemiology, and End Results
TNBC: triple negative breast cancer

## References

[1] DeSantis CE, Fedewa SA, Goding Sauer A, et al. Breast cancer statistics, 2015: Convergence of incidence rates between black and white women. CA Cancer J Clin 2016;66:31–42. 26513636 10.3322/caac.21320

[2] Osei-Twum JA, Gedleh S, Lofters A, et al. Differences in Breast Cancer Presentation at Time of Diagnosis for Black and White Women in High Resource Settings. J Immigr Minor Health 2021;23:1305–1342. 33721146 10.1007/s10903-021-01161-3 PMC8599379

[3] DeSantis CE, Ma J, Gaudet MM, et al. Breast cancer statistics, 2019. CA Cancer J Clin 2019;69:438–451. 31577379 10.3322/caac.21583

[4] DeSantis CE, Miller KD, Goding Sauer A, et al. Cancer statistics for African Americans, 2019. CA Cancer J Clin 2019;69:211–233. 30762872 10.3322/caac.21555

[5] Martini R, Newman L, Davis M. Breast cancer disparities in outcomes; unmasking biological determinants associated with racial and genetic diversity. Clin Exp Metastasis 2021. 10.1007/s10585-021-10087-x PMC9337912 33950410

[6] Daly B, Olopade OI. A perfect storm: How tumor biology, genomics, and health care delivery patterns collide to create a racial survival disparity in breast cancer and proposed interventions for change. CA Cancer J Clin 2015;65:221–238. 25960198 10.3322/caac.21271

[7] Sighoko D, Murphy AM, Irizarry B, et al. Changes in the racial disparity in breast cancer mortality in the ten US cities with the largest African American populations from 1999 to 2013: The reduction in breast cancer mortality disparity in Chicago. Cancer Causes Control 2017;28:563–568. 28275936 10.1007/s10552-017-0878-y PMC5400784

[8] Chu KC, Lamar CA, Freeman HP. Racial disparities in breast carcinoma survival rates: Seperating factors that affect diagnosis from factors that affect treatment. Cancer 2003;97:2853–2860. 12767100 10.1002/cncr.11411

[9] Wojcik BE, Spinks MK, Optenberg SA. Breast carcinoma survival analysis for African American and White women in an equal-access health care system. Cancer 1998;82:1310–1318. 9529023 10.1002/(sici)1097-0142(19980401)82:7<1310::aid-cncr14>3.0.co;2-9

[10] Jatoi I, Becher H, Leake CR. Widening disparity in survival between White and African-American patients with breast carcinoma treated in the US Department of Defense healthcare system. Cancer 2003;98:894–899. 12942554 10.1002/cncr.11604

[11] Rizzo JA, Sherman WE, Arciero CA. Racial disparity in survival from early breast cancer in the department of defense healthcare system. J Surg Oncol 2015;111:819–823. 25711959 10.1002/jso.23884

[12] Hortobagyi GN, Edge SB, Giuliano A. New and Important Changes in the TNM Staging System for Breast Cancer. Am Soc Clin Oncol Educ Book 2018;38:457–467. 30231399 10.1200/EDBK_201313

[13] Bloom HJ, Richardson WW. Histological grading and prognosis in breast cancer; a study of 1409 cases of which 359 have been followed for 15 years. Br J Cancer 1957;11:359–377. 13499785 10.1038/bjc.1957.43 PMC2073885

[14] Elston CW, Ellis IO. Pathological prognostic factors in breast cancer. I. The value of histological grade in breast cancer: Experience from a large study with long-term follow-up. Histopathology 1991;19:403–410. 1757079 10.1111/j.1365-2559.1991.tb00229.x

[15] Hammond MEH, Hayes DF, Dowsett M, et al. American Society of Clinical Oncology/College of American Pathologists guideline recommendations for immunohistochemical testing of estrogen and progesterone receptors in breast cancer. J Clin Oncol 2010;28:2784–2795. 20404251 10.1200/JCO.2009.25.6529 PMC2881855

[16] Wolff AC, Hammond MEH, Allison KH, et al. Human Epidermal Growth Factor Receptor 2 Testing in Breast Cancer: American Society of Clinical Oncology/College of American Pathologists Clinical Practice Guideline Focused Update. J Clin Oncol 2018;36:2105–2122. 29846122 10.1200/JCO.2018.77.8738

[17] Newman LA, Griffith KA, Jatoi I, et al. Meta-analysis of survival in African American and white American patients with breast cancer: Ethnicity compared with socioeconomic status. J Clin Oncol 2006;24:1342–1349. 16549828 10.1200/JCO.2005.03.3472

[18] Foy KC, Fisher JL, Lustberg MB, et al. Disparities in breast cancer tumor characteristics, treatment, time to treatment, and survival probability among African American and white women. NPJ Breast Cancer 2018;4:7. 29582015 10.1038/s41523-018-0059-5 PMC5861087

[19] Hines RB, Johnson AM, Lee E, et al. Trends in Breast Cancer Survival by Race-Ethnicity in Florida, 1990-2015. Cancer Epidemiol Biomarkers Prev 2021;30:1408–1415. 34210675 10.1158/1055-9965.EPI-20-1746

[20] Danforth DN Jr. Disparities in breast cancer outcomes between Caucasian and African American women: A model for describing the relationship of biological and nonbiological factors. Breast Cancer Res 2013;15:208. 23826992 10.1186/bcr3429 PMC3706895

[21] Marlow NM, Pavluck AL, Bian J, et al. The Relationship Between Insurance Coverage and Cancer Care: A Literature Synthesis. Research Triangle Park (NC), 2009. 31216155

[22] Jemal A, Robbins AS, Lin CC, et al. Factors That Contributed to Black-White Disparities in Survival Among Nonelderly Women With Breast Cancer Between 2004 and 2013. J Clin Oncol 2018;36:14–24. 29035645 10.1200/JCO.2017.73.7932

[23] Short LJ, Fisher MD, Wahl PM, et al. Disparities in medical care among commercially insured patients with newly diagnosed breast cancer: Opportunities for intervention. Cancer 2010;116:193–202. 19877115 10.1002/cncr.24691

[24] Semprini J, Olopade O. Evaluating the Effect of Medicaid Expansion on Black/White Breast Cancer Mortality Disparities: A Difference-in-Difference Analysis. JCO Glob Oncol 2020;6:1178–1183. 32721196 10.1200/GO.20.00068 PMC7392753

[25] Cooper RM, Chung J, Hogan T, et al. Influence of health care systems on mortality in adult patients with cancer. Am J Manag Care 2021;27:182–185. 34002959 10.37765/ajmc.2021.88631

[26] Haque R, Xu X, Shi J, et al. Breast Cancer Outcomes in a Racially and Ethnically Diverse Cohort of Insured Women. Ethn Dis 2018;28:565–574. 30405302 10.18865/ed.28.4.565 PMC6200302

[27] Enewold L, Zhou J, McGlynn KA, et al. Racial variation in breast cancer treatment among Department of Defense beneficiaries. Cancer 2012;118:812–820. 21766298 10.1002/cncr.26346 PMC4123115

[28] Eaglehouse YL, Georg MW, Shriver CD, et al. Racial Differences in Time to Breast Cancer Surgery and Overall Survival in the US Military Health System. JAMA Surg 2019;154:e185113. 30673075 10.1001/jamasurg.2018.5113 PMC6439631

[29] Lovejoy LA, Eaglehouse YL, Hueman MT, et al. Evaluation of Surgical Disparities Between African American and European American Women Treated for Breast Cancer Within an Equal-Access Military Hospital. Ann Surg Oncol 2019;26:3838–3845. 31410609 10.1245/s10434-019-07706-z

[30] Vargason AB, Turner CE, Shriver CD, et al. Genetic testing in Non-Hispanic Black women with breast cancer treated within an equal-access healthcare system. Genet Med 2022;24:232–237. 34906450 10.1016/j.gim.2021.08.002

[31] Jayasekara H, MacInnis RJ, Chamberlain JA, et al. Mortality after breast cancer as a function of time since diagnosis by estrogen receptor status and age at diagnosis. Int J Cancer 2019;145:3207–3217. 30771221 10.1002/ijc.32214 PMC6697632

[32] Pan H, Gray R, Braybrooke J, et al. 20-Year Risks of Breast-Cancer Recurrence after Stopping Endocrine Therapy at 5 Years. N Engl J Med 2017;377:1836–1846. 29117498 10.1056/NEJMoa1701830 PMC5734609

